# Detection of residual pulmonary alterations with lung ultrasound and effects on postoperative pulmonary complications for patients with asymptomatic SARS-CoV-2 infection undergoing surgeries

**DOI:** 10.1186/s12871-022-01715-4

**Published:** 2022-06-16

**Authors:** Susana González-Suárez, Antonio Barbara Ferreras, Melissa Caicedo Toro, Macarena Aznar de Legarra

**Affiliations:** 1 Department of Anesthesiology, Hospital Universitari Vall d’Hebron, Passeig de la Vall d’Hebron 119, 08035, Barcelona, Spain; 2 Universitat Autònoma de Barcelona, UAB, Barcelona, Spain

**Keywords:** Post-COVID-19 patients, Lung ultrasound, Static compliance, Postoperative pulmonary complications

## Abstract

**Background:**

For patients with a clinical course of active SARS-CoV-2 (severe acute respiratory syndrome coronavirus 2) infection, there may be a higher risk of perioperative complications. Our main objective is to detect the residual pulmonary alterations in asymptomatic patients after SARS-CoV-2 infection undergoing surgery and determine their relationship with the clinical course of SARS-CoV-2 infection. The secondary aim is to investigate whether the presence of residual pulmonary alterations have any affects on the severity of postoperative pulmonary complications.

**Methods:**

After approval by the Hospital’s Ethical Committee, this prospective observational study included consecutive patients (*n*=103) undergoing various surgical procedures and anesthetic techniques with a history of past SARS-CoV-2 infection. On the day of surgery these patients remained asymptomatic and the polymerase chain reaction (PCR) test for SARS-CoV-2 was negative. The history, physical findings, and clinical course of SARS-CoV-2 infection were recorded. Lung ultrasound was performed before surgery to evaluate the possible residual pulmonary alterations (≥ 3 B-lines and pleural thickening), along with determitation of pulmonary static compliance values during surgery. Postoperative pulmonary complications were collected during hospital stay.

**Results:**

24.27% (*n*=25) patients presented ≥ 3 B-lines, and 28% (*n*=29) patients presented pleural thickening. For 15 patients (21.7%) the pulmonary compliance was < 40 mL/cm H_2_O**.** Patients with pleural thickening had a higher incidence of pneumonia, acute respiratory syndrome distress, a need for vasoactive drugs and required more days of hospitalization during SARS-CoV-2 infection (*p=* 0.004, 0.001, 0.03, 0.00 respectively). Patients with ≥ 3 B-lines needed more days in an intensive care unit and vasoactive drugs during SARS-CoV2 infection (*p*= 0.04, 0.004 respectively). Postoperative pulmonary complications were observed in 5.8% (*n*=6) of the patients, and were more frequent in the presence of both, ≥ 3 B-lines and pleural thickening (*p*= 0.01).

**Conclusions:**

In asymptomatic post-COVID-19 patients, pathological findings detected by lung ultrasound before surgery are associated with the severity of the SARS-CoV2 infection and resulted in more postoperative pulmonary complications. In these patients, the incidence of postoperative pulmonary complications appears similar to that described in the surgical population before the pandemic.

**Trial registration:**

clinicaltrials.gov (NCT04922931). June 21, 2021. “Retrospectively registered”

## Background

The coronavirus disease 2019 (COVID-19), caused by a coronavirus SARS-CoV-2, infects a large part of our population. In surgical patient’s COVID-19 can cause a quick deterioration of lung function [1–3]. In addition to a lung disorder, an alteration in other organs can occur and increase mortality [4–6].

Based on clinical information and expert recommendations, it is suggested that for patients with a possible COVID-19 infection, elective surgeries may be canceled or postponed with the focus to only maintain emergency operations and elective cancer surgeries [4–10]. Some authors obtained that the time of surgery for a patient who has suffered from SARS-CoV-2 should be delayed up to at least 7 weeks after passing the infection [11].

To reduce the mortality rate and postoperative pulmonary complications (PPC) we have included the patients who underwent surgery on post-COVID-19 patients when the infection had disappeared, there were no symptoms, and the laboratory testing for SARS-CoV-2 infection was negative. In these circumstances, since the patients included in our study suffered from the disease, residual lung lesions could be found despite being asymptomatic. Moreover, a lower incidence of PPC than that described in patients with active SARS-CoV-2 infection at the time of surgery could be expected.

## Methods

### Ethics statement and registration

The Ethics Committee approved this prospective observational single-center study at the Vall d’Hebron University Hospital, Barcelona, Spain (PR (AG)346/2020) and retrospective registered at www.clinicaltrials.gov on June 11, 2021 (registration number: NCT04922931). All participants signed written informed consent forms before enrolment in the study. All methods were carried out following relevant guidelines and regulations.

### Study design and participants

We defined post-COVID-19 patient as a patient who previously had a positive PCR test from oronasopharyngeal swab, and then had one negative test performed at least 4 days before surgery and the absence of symptoms (fever, dyspnea, cough, and digestive disorders) at time of surgery.

We included all consecutive post-COVID-19 patients ≥ 18 years underwent various elective surgery (general surgery, urology, neurology, otolaringology, gynecology, cardiac, thoracic, and vascular surgery) and anesthetic techniques (general, local, neuraxial and peripheral regional anesthesia) from June 30, 2020, to February 18, 2021. Moreover, the included patients did not have baseline pulmonary pathology prior to COVID-19.

**Fig. 1:**
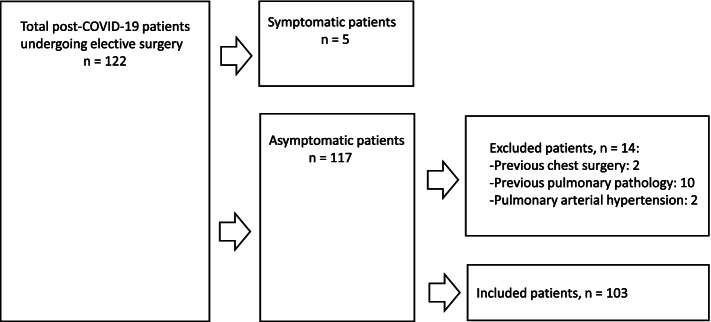
Flow diagram of exclusion/inclusion criteria

Exclusion criteria were as follows: patients under the age of 18 years old, pregnant, patients with hemodynamic instability parameters (mean arterial pressure < 60 mmHg and need for vasopressors), patients undergoing pulmonary surgery (lung neoplasms) or who previously had chest surgery, patients with pulmonary pathology before SARS-CoV-2 infection (obstructive or restrictive pulmonary pathology), patients with a documented medical history of any degree of pulmonary arterial hypertension (both primary or secondary), heart failure or active respiratory infection. Figure 1 shows the flow diagram of the inclusion/exclusion process.

### Pulmonary ultrasonography procedure and determination of pulmonary static compliance

The anesthesiologists used the Sonosite portable ultrasound system and a 2- to 5 MHz convex transducer for lung ultrasound (LUS) procedure. During study researchers utilized higher frequencies for evaluation of the pleural line to determine pleural thickening (defined as being greater than 3 mm in width, with or without irregular margins) [12] as well as a linear probe 6-15MHz if it was deemed necessary. The scanning system included the approach of 10 thoracic areas with 5 in each hemithorax. Specifically, areas 1 and 2 denoted the upper anterior and lower anterior chest areas respectively; areas 3 and 4 denoted the upper lateral and basal lateral chest areas, respectively, and area 5 denoted posterior area located posterior of the axillary line, at the point at which the diaphragm and adjacent lung segment were accessible in a supine position with slight lateralization of the patient. The anterolateral areas were delimited by three longitudinal lines: para-sternal line, anterior axillary line, and posterior axillary line. A breast line delimited the upper and lower areas resulting in five punctures of exploration (modified from Volpicelli et al. [13].

During the LUS procedure, the probe was placed vertically perpendicular to the ribs at a depth of 9-12 cm in the anterior plane and 12-20 cm in the lateral plane, and the focus was placed at the pleural level.

During operation, the static compliance was determined by respirators (Drager Perseus A500 and MAQUET ventilator) in patients with general anesthesia. The controlled ventilation was performed with tidal volumes of 7ml/kg adjusted for ideal body weight, with a PEEP (Positive End-Expiratory Pressure) of 5 mm Hg and in a neuromuscular blocking state after orotracheal intubation in a supine position. The static compliance value was calculated depending on the formula that is available in anesthesiology textbook [14] as follows: Cs = VT/P *plateau* – PEEP where Cs denotes static compliance, VT denotes tidal volume, and P plateau denotes plateau pressure.

### Study outcomes

We used as the main outcome the proportion of patients having the residual pulmonary alterations detected by lung ultrasound and static pulmonary compliance before operation, and the relationship between these injuries with the clinical course of SARS-CoV-2 infection (cough, dyspnea, fever, digestive disorders, pneumonia, thrombotic-associated complications (PTE, myocardial infarction, stroke, venous thrombosis), ARDS, need for mechanical ventilation, need for vasoactive drugs and days of hospitalization and admission to Intensive Care Unit).

The secondary outcomes were the postoperative occurrence of respiratory complications during hospital stay (ARDS, PTE, pneumonia, pneumothorax, atelectasis, bronchospasm) and determine whether the incidence of residual pulmonary injuries detected before surgery are associated with the appearance of PPC.

Other secondary outcome measures were the correlation between static pulmonary compliance and B-lines and correlation between lung compliance values and the time elapsed between COVID-19 diagnosis and surgery. Other outcomes for the postoperative period were 30-day survival after surgery; hospital stay; need for IMV and NIMV; need for an emergent intubation [15]. Other outcomes for the postoperative complications were: the influence of the ASA, surgical complexity and type of anesthesia on the incidence of PPC, occurrence of postoperative non-pulmonary complications (PnPC) (need for vasoactive drugs, transfusion requirements, need for a second surgery, thrombotic-associated complications (upper or lower extremity ischemia, intestinal ischemia, acute myocardial infarction and stroke), non-pulmonary infections, acute kidney injury, arrhythmias and heart failure).

### Data collection

We collected demographic characteristics, baseline comorbidities, American Society of Anesthesiologists (ASA) physical status classification, baseline characteristics of the COVID-19 presentation and the number of days from COVID-19 diagnosis to surgery.

LUS data collection comprised the presence of atelectasis, pleural effusion, B lines, pleural thickening, and the presence of both, ≥ 3 B-lines and pleural thickening.

The operative data collected included the static pulmonary compliance values in patients who received general anesthesia, the type of anesthesia performed, the type of surgery, and the risk of surgery according to the National Institute for Clinical Excellence (NICE) classification of the National Health Service UK.

Postoperative data collected included 30-day survival after surgery, hospital stay, need for IMV and NIMV, emergent intubation, type of PnPC and type of PPC.

All items that could be used to identify the patient (clinical record ID number or name) were removed to protect personal data.

### Data measurement

For LUS, each region was scored according to four LUS aeration patterns inspired by Lichtenstein’s nomenclature [16]: 0 points—presence of lung sliding with A-lines or one or two isolated B-lines; 1 point—moderate loss of lung aeration with three or four B- lines; 2 points—severe loss of lung aeration with five or more B-lines; and 3 points—presence of a hypoechoic poorly defined tissue characterized by complete loss of lung aeration. The LUS score ranging between 0 and 30 was calculated as the sum of points.

For surgical risk, we classified the surgical procedures according to the following grades:

I. I: Minor surgical procedures: poor surgical aggressiveness, surgery with low probability of bleeding or easily detectable bleeding (excision of adenopathies, herniorrhaphys, amputation of fingers, lithotripsy, prostate biopsy).
II. II: Medium surgical procedures: greater probability of bleeding and/or if it occurs could go unnoticed by developing in a cavity (thyroidectomy, embolectomy, tonsillectomy, transurethral resection, tracheostomy, laparoscopy surgery for general, ginecology and urology surgeries).
III. III: Major surgical procedures: most important degree of surgical aggressiveness with prolonged postoperative need (peripheral bypass-bypass, spinal arthrodesis, laminectomy, open surgery (for resection of the digestive tract, cystectomy, nephrectomy).
IV. IV: Very aggressive surgical procedures: prolonged surgeries with very specialized or critical care in the postoperative period, (open cardiac surgery, aortic surgery, intracranial surgery, aggressive neoplastic surgery (pelviperitonectomy).

We used the following definitions for PCP:

- ARDS detected by hypoxemia with a relationship between the arterial partial pressure of oxygen and the fraction on inspired oxygen (PaO2/FiO2) less than 200, and bilateral pulmonary infiltrates in both fields on a chest radiograph.
- PTE was diagnosed by dyspnea clinic and chest computed tomography angiography (CT angiography).
- Pneumonia when the patient received antibiotics for a suspected respiratory infection and met at least one of the following criteria: fever, new lung opacities, leukocyte count >12.000/μ.
- Atelectasis when the patient presented lung opacification with a shift of the hilum, hemidiaphragm, or mediastinum toward the affected area on a chest radiograph.
- Bronchospasm, when the patient presented expiratory wheezing, treated with bronchodilators.
- Pneumothorax, when the patient presented air in the pleural space on a chest radiograph.

The PnPC were recorded as reported by the attending physicians.

### Data sources and management

Data was either collected prospectively by the research team (ultrasound findings and pulmonary static compliance) and retrospectively by the research team (clinical course of SARS-CoV-2 infection and postoperative data). Retrospective data were obtained from the patient’s medical records.

### Statistical analysis

For categorical variables, frequencies and percentages were displayed for the total sample. Differences in parameters were calculated with *Pearson's Chi-squared* non-parametric test. The mean, standard deviation, and percentile descriptive were displayed for continuous variables. Differences in parameters were evaluated by Mann-Whitney (non-parametric) test or *Student’s t tests* for two independent (parametric) samples based on the normality of the variables to be tested using the *Shapiro-Wilks test*. *Spearman's correlation* coefficient was employed to measure the correlation between two variables. The level of significance used in the analyses was 5% (*α-0.05*) for two- tailed tests.

## Results

One hundred three post-COVID-19 patients were included in the study. The average number of days between COVID-19 diagnosis and surgery was 108.56 ± 82.02. The mean age was 60.18 ± 14.95 years. During SARS-CoV-2 infection, the number of days of admission to the ICU was 2.09 ± 8.82, IQR (0-1) and the total days of hospitalization were 14.43 ± 44.69, IQR (2-8). The commorbidities of the patients and their relationship with the pathological ultrasonographic findings and static compliance are shown in Table 1. Surgical and anesthetic characteristics are shown in Table 2.

**Table 1:** Demographic characteristics, comorbidities, and their association with pulmonary pathological ultrasonographic findings and static compliance

	Total patients ***n*** = 103	Pleural thickening, ***n*** = 29 (total ***n*** = 103)	***p-value***	B-lines ≥ 3, ***n*** = 25 (total ***n*** = 103)	***p-value***	Compliance <50, ***n*** = 35 (total ***n*** = 69)	***p-value***
**Age**							
**<50**	23 (22.3)	1 (3.4)	0.005	3 (12)	0.33	6 (17.1)	0.93
**50-69**	43 (41.7)	12 (41.4)		11 (44)		18 (51.4)	
**≥70**	37 (35.9)	16 (55.2)		11 (44)		11 (31.4)	
**Sex**							
**Woman**	45 (43.7)	8 (27.6)	0.04	8 (32)	0.33	20 (57.1)	0.18
**Man**	58 (56.3)	21 (72.4)		17 (68)		15 (42.9)	
**ASA**							
**I**	7 (6.8)	0 (0)	0.12	0 (0)	0.08	2 (5.7)	0.48
**II**	55 (53.4)	13 (44.8)		10 (40)		21 (60)	
**III**	38 (36.9)	15 (51.7)		14 (56)		10 (28.6)	
**IV**	3 (2.9)	1 (3.4)		1 (4)		2 (5.7)	
**Cardiopathy**	27 (26.2)	11 (37.9)	0.23	10 (40)	0.18	12 (34.3)	0.43
**Vascular disease**	14 (13.6)	5 (17.2)	0.49	3 (12)	0.79	7 (20)	0.08
**Renal disease**	14 (13.6)	4 (13.8)	0.97	3 (12)	0.79	2 (5.7)	0.57
**Hypertension**	61 (59.2)	20 (69)	0.21	15 (60)	0.93	21 (60)	0.68
**Diabetes mellitus**	32 (31.1)	10 (34.4)	0.89	8 (32)	0.90	8 (22.9)	0.72

Data are expressed as number (percentage

**Table 2:** Anesthetic-surgical characteristics

Total patients, *n* =103		
Surgery	General	42 (40.8)
	Urology	21 (20.4)
	Vascular	9 (8.7)
	Neurology	7 (6.8)
	Cardiac	11 (10.7)
	Thoracic	5 (4.9)
	Otolaryngology	4 (3.9)
	Gynecology	4 (3.9)
Surgical complexity	I	14 (13.6)
	II	44 (42.7)
	III	32 (31.1)
	IV	13 (12.6)
Type of anesthesia	General	69 (66.9)
	Spinal anesthesia	22 (21.3)
	Peripheral nerve block	3 (2.9)
	Local anesthesia	9 (8.7)

Data are expressed as number (percentage)

At the time of surgery, 11.7% of the patients presented atelectasis, and 7.8% presented pleural effusion. The presence of ≥ 3 B-lines were observed in 25 (24%) of the patients. The global mean of B-lines score was 0.71 ± 1.56, IQR (0-0), and in patients with ≥ 3 B- lines was 2.84 ± 1.82, IQR (0-4). Sixteen patients (15.53%) presenting both, B-lines ≥ 3 and pleural thickening.

**Fig. 2:**
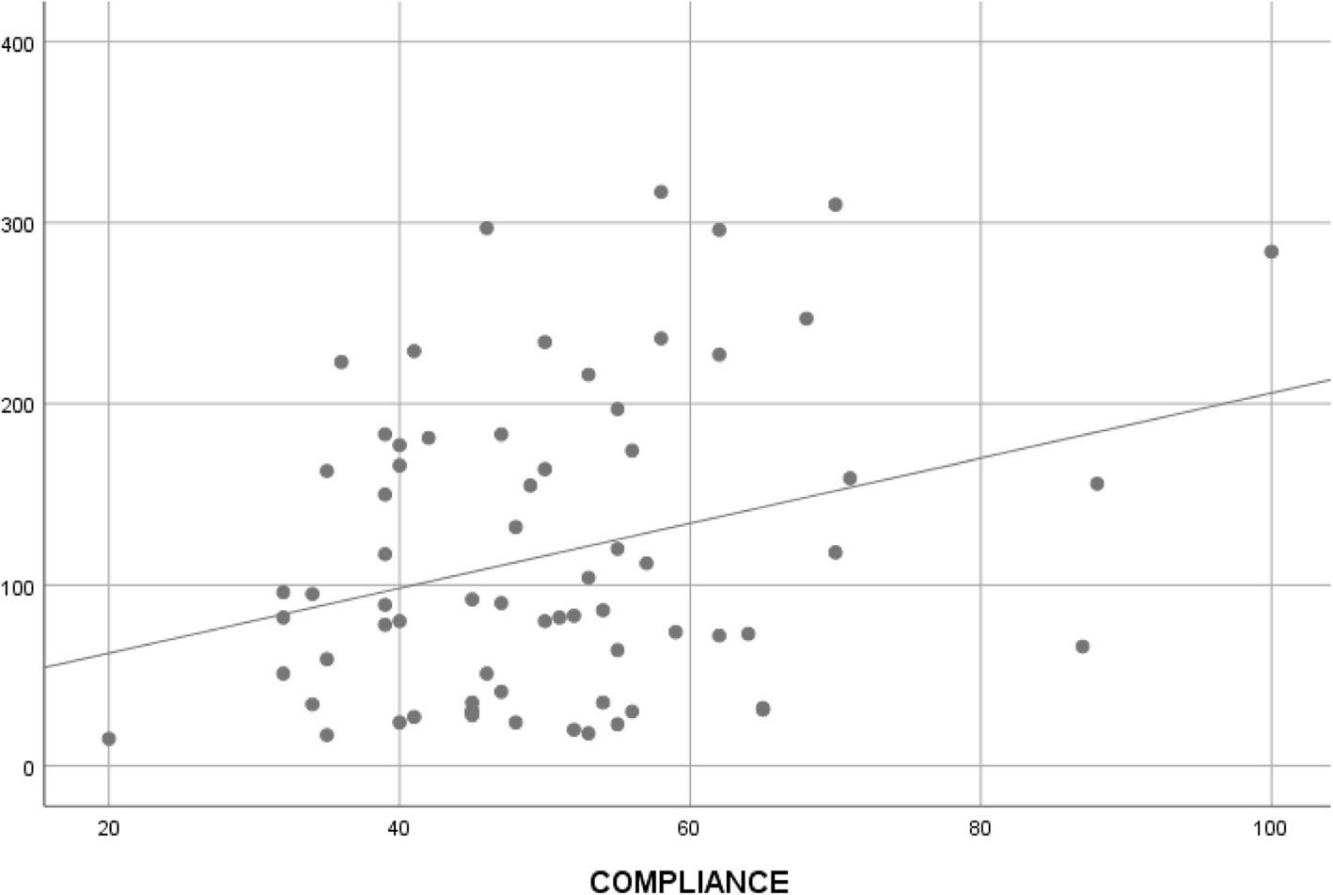
Correlation between pulmonary static compliance and time elapsed from COVID diagnostic to surgery

**Fig. 3:**
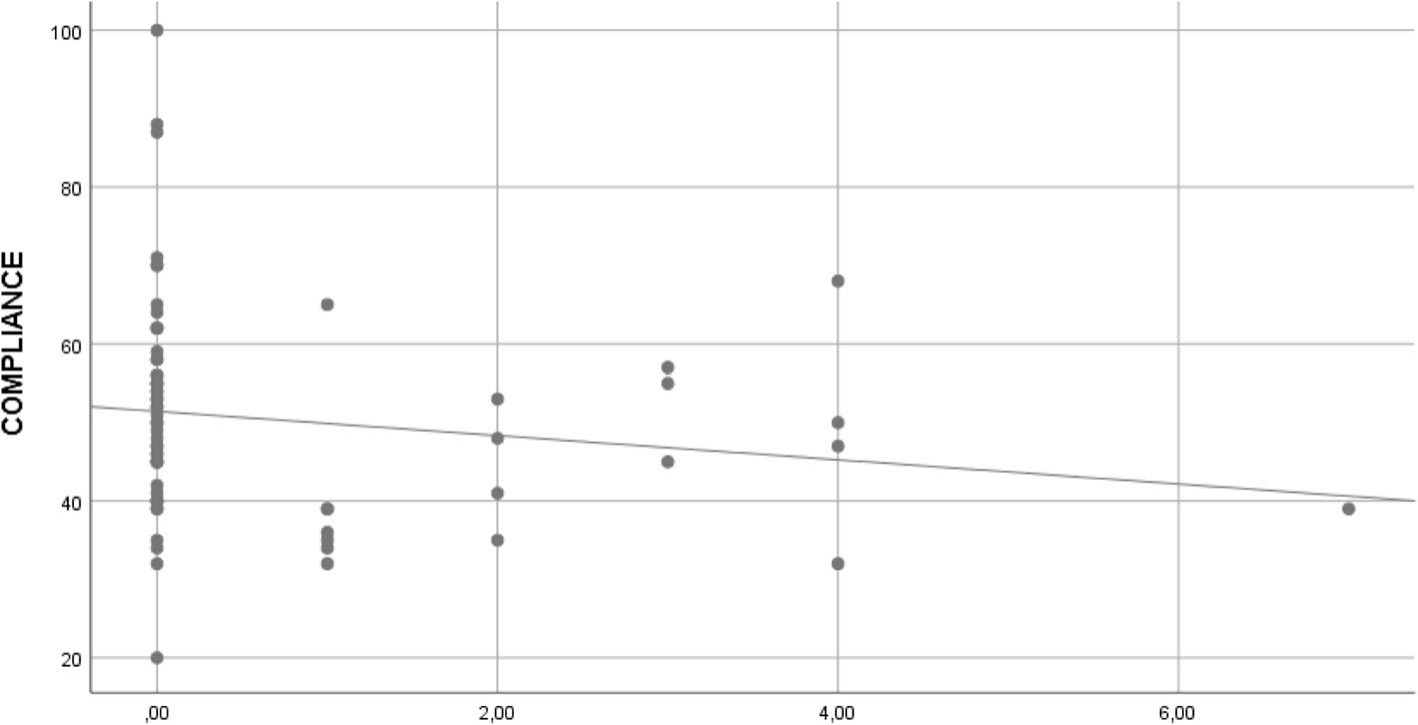
Correlation between pulmonary static compliance and B lines

The global mean of pulmonary compliance obtained in 69 patients who required general anesthesia, was 50.39 ± 13.89 ml/cm H_2_O. For 15 patients (21.7%) the pulmonary compliance was < 40; for 20 patients (29%) it was between 40-49, and for 34 patients (49.2%) it was ≥ 50 ml/cm H_2_O. The pulmonary compliance values were higher as the time elapsed between COVID-19 diagnosis and surgery increased (*rho=* 0.24, *p*=0.04) (Fig. 2). An inverse correlation was seen between pulmonary compliance and B lines (Fig. 3) (*rho=* -0.24, *p*=0.04).

The patients with pleural thickening presented more hospitalization days, a need for mechanical ventilation, more incidence of dyspnea, fever, pneumonia, SDRA and a need for vasoactive drugs during SARS-CoV2 infection (Table 3).

**Table 3:** Abnormal pulmonary ultrasonographic findings and clinical course of the SARS-CoV-2 infection

Pleural thickening				B-lines (≥ 3)		
	No, 74 (71.84)	Yes, 29 (28.15)	*p-value*	No, 78 (75.72)	Yes, 25 (24.27)	*p-value*
**Covid19-Surgery**	109 ± 84.31	106.93 ± 77.37	0.33	115.23 ± 84.90	88 ± 70.03	0.15
**Hospitalization days**	2.65 ± 6.85	12.90 ±26.35	0.000	3.28 ± 7.19	12.56 ±28.46	0.11
**ICU days**	0.34 ±1.46	6.24 ±17.28	0.01	0.72 ±3.68	6.00 ±17.88	0.04
**NIMV days**	0.23 ± 1.58	1.34 ± 3.02	0.002	0.36 ± 1.84	1.12 ± 2.82	0.42
**IMV days**	0.09 ± 0.81	4.34 ± 13.65	0.03	0.41 ± 2.93	4.04 ± 14.04	0.06
**Cough**	22 (29.7)	10 (34.5)	0.64	24 (30.8)	8 (32.0)	0.91
**Dyspnea**	14 (18.9)	14 (48.3)	0.003	19 (24.4)	9 (36.0)	0.25
**Fever**	24 (32.4)	16 (55.2)	0.03	30 (38.5)	10 (40.0)	0.89
**Digestive disorders**	13 (17.6)	7 (24.1)	0.45	15 (19.2)	5 (20.0)	0.93
**Pneumonia**	19 (25.7)	16 (55.2)	0.004	24 (30.8)	11 (44.0)	0.22
**Venous thrombosis**	0 (0.0)	1 (3.4)	0.11	0 (0.0)	1 (4.0)	0.07
**PTE**	1 (1.4)	0 (13.8)	0.53	1 (1.3)	0 (0.0)	0.53
**ARDS**	0 (0.0)	4 (34.5)	0.001	2 (2.6)	2 (8.0)	0.01
**Vasoactive drugs**	1 (1.4)	3 (10.3)	0.02	1 (1.3)	3 (12.0)	0.01

COVID-19-Surgery: Days elapsed from diagnosis of SARS-CoV-2 infection to the day of surgery. *ICU* Intensive Care Unit. *NIMV* Non-invasive mechanical ventilation, *IMV* Invasive mechanical ventilation, *PTE* Pulmonary thromboembolism, *ARDS* Acute respiratory distress syndrome. Data are expressed as mean ± SD, and number (percentage)

The patients with ≥ 3 B-lines had a higher incidence of ARDS, and they needed more days in intensive care unit (ICU) and vasoactive drugs during SARS-CoV-2 infection (Table 3).

There was no association between compliance < 50 ml/cmH2O and clinical course of COVID-19 infection (Table 4).

**Table 4:** Static compliance and clinical course of the SARS-CoV-2 infection

Compliance			
	<50, *n* =35	≥50, *n* =34	*p-value*
**Covid19-Surgery**	101.34 ± 73.16	135.03 ± 92.80	0.14
**Hospitalization days**	5.03 ± 14.87	5.82 ± 9.70	0.24
**ICU days**	2.52 ± 9.30	1.29 ± 5.39	0.32
**NIMV days**	0.94 ± 2.89	0.35 ± 1.25	0.64
**IMV days**	1.51 ± 7.83	0.74 ± 4.29	0.57
**Cough**	13 (37.1)	13 (38.2)	0.92
**Dyspnea**	9 (25.7)	12 (35.3)	0.38
**Fever**	11 (31.4)	18 (52.9)	0.07
**Digestive disorders**	9 (25.7)	9 (26.5)	0.94
**Pneumonia**	12 (34.3)	15 (44.1)	0.40
**Venous thrombosis**	1 (2.9)	0 (0)	0.32
**PTE**	1 (2.9)	0 (0)	0.32
**ARDS**	2 (5.7)	1 (2.9)	0.57
**Vasoactive drugs**	3 (8.6)	0 (0)	0.08

COVID-19-Surgery: Days elapsed from diagnosis of SARS-CoV-2 infection to the day of surgery. *ICU* Intensive Care Unit. *NIMV* Non-invasive mechanical ventilation, *IMV* Invasive mechanical ventilation, *PTE* Pulmonary thromboembolism, *ARDS* Acute respiratory distress syndrome. Data are expressed as mean ± SD, and number (percentage)

All patients survived 30 days after surgery. Hospital stay was 5.53 ± 15.67 days. The mean time for IMV was 1.29 ± 7.44, and for NIMV was 0.54 ± 2.13 days. Two patients presented emergent orotracheal intubation due to PPC.

Six patients (5.8%) presented PPC. In these patients, the mean age was 56.50 ± 17.97, one woman and five men (*p* = 0.17)). One patient was ASA II, four patients were ASA III, and one patient ASA IV (*p* = 0.05). Three patients presented a degree of type 2 surgical complexity, one patient presented type 3 and two patients presented type 4 (*p* = 0.32). Five patients with PPC received general anesthesia and one patient endovenous sedation (*p=* 0.64).

The patients presented six different types of PPC. Two of these patients required emergent orotracheal intubation; whilst only one patient presented several complications simultaneously: PTE, pneumonia, pneumothorax, and bronchospasm, with the need for IMV for 30 days and NIMV for 4 days. Moreover, one patient presented pneumonia, one patient presented bronchospasm, and one patient presented ARDS with the need for IMV for 7 days. Two patients presented atelectasis, one of which required NIMV for 4 days.

PPC and their relationship with pulmonary echographic findings and static compliance are shown in Table 5. Patients with both, ≥ 3 B-lines and pleural thickening, presented a higher occurrence of PPC.

**Table 5:** Postoperative pulmonary complications and their relationship with pulmonary compliance, B-lines, and pleural thickening

			Postoperative pulmonary complications		
			None	Some	***p-value***
**Pulmonary compliance**	Total	69 (100)	64 (100)	5 (100)	0.66
	<50	35 (50.7)	32 (50)	3 (60)	
	≥50	34 (49.3)	32 (50)	2 (40)	
**B-lines**	Total	103 (100)	97 (100)	6 (100)	0.13
	<3	78 (75.7)	75 (77.3)	3 (50)	
	≥3	25 (24.3)	22 (22.7)	3 (50)	
**Pleural thickening**	Total	103 (100)	97 (100)	6 (100)	0.22
	No	74 (71.8)	71 (73.2)	3 (50)	
	Yes	29 (28.2)	26 (26.8)	3 (50)	
**B-lines + Pleural thickening**	Total	103 (100)	97 (100)	6 (100)	0.01
	No	87 (84.5)	84 (86.6)	3 (50)	
	Yes	16 (15.5)	13 (13.4)	3 (50)	

Data are expressed as number (percentage)

The PnPC were gathered: 4 (3.9%) patients needed vasoactive drugs, 4 (3.9%) patients required a second surgery, 2 (1.9%) patients presented thrombosis, 3 (2.9%) patients presented surgical wound infection, 2 (1.9%) patients presented cardiac alteration and 1 (1%) patient presented renal alterations. 15 patients (14.56%) required red blood cell transfusion and 2 patients (1.94%) required fresh frozen plasma and platelets.

## Discussion

In asymptomatic post-COVID-19 patients there are residual lung lesions that are related to the clinical course of SARS-CoV-2 infection. Patients with pleural thickening at the time of surgery had more symptoms of dyspnea, more days of hospitalization and noninvasive/invasive mechanical ventilation during SARS-CoV-2 infection comparted to patients who did not have pleural thickening (*p*= 0.003, 0.000, 0.002, 0.03 respectively). Also, patients with pleural thickening presented a clinical course with a higher incidence of pneumonia, acute respiratory syndrome distress, and a need for vasoactive drugs during SARS-CoV2 infection (*p*= 0.004, 0.001, 0.03 respectively). Patients with ≥ 3 B-lines needed more days in intensive care unit and needed vasoactive drugs during SARS-CoV2 infection (*p*= 0.04. 0.004). No signs of symptoms, days of hospitalization, or clinical course of COVID-19 were observed in patients with compliance < 50 ml/cmH_2_O the day of surgery.

Other authors also demonstrated the presence of residual lung wounds by SARS-CoV-2 infection. Lombardi F. et al. [17] and Frija-Masson J. et al. [18] showed that the residual respiratory impairment, including lower exercise tolerance, was correlated with the severity of respiratory failure during hospitalization. Abdulrahman et al. [19] and Myall et al. [20] showed that although most cases of COVID-19 recover completely, a small proportion of patients present pathological pulmonary findings by LUS with the appearances of pleural line abnormalities and B line artifacts, which result from inflammation and interstitial thickening that increase in number with severity. The persistence of LUS abnormalities was also seen in patients with dyspnea [21]; our study also found that dyspnea is correlated with an increase in pulmonary pathological findings by LUS.

However, we observed no correlation between the severity of the SARS-CoV-2 infection and a decrease in static pulmonary compliance. It is probable that the time between the diagnosis of the SARS-CoV-2 infection and surgery had been sufficient time to cure the infection. Thus, we observed that the pulmonary compliance values were higher as the time elapsed between COVID-19 diagnosis and surgery (Fig. 2).

Some patients in our study obtained low values of pulmonary compliance (< 40 ml/cm H_2_O in 21.7 % patients); in some of these patients, this low compliance could be associated with the presence of pulmonary “fibrosis” [22–24]. This fibrosis has been shown after isolated viral infections [25] and especially after persistent viral infections [26]. Nevertheless, Wallace et al. [27] argues that ¨fibrosis¨ should not apply to the abnormalities seen in cases of post-viral pulmonary fibrosis, since these changes could be reversed over time. Some authors argue that ¨fibrosis¨ can be considered a potentially reversible process, and the term ¨reversible pulmonary fibrosis¨ has been used in the current literature [28]. Truly, this term encompasses a non-idiopathic form of pulmonary fibrosis associated with COVID-19 infection, which is heterogeneous in many aspects and can present anytime from initial hospitalization to long term follow-up. However, there is still much uncertainty about many aspects of the COVID-19 condition.

On the other hand, we demonstrated an inverse correlation between pulmonary compliance and B-lines. Other authors also argue that the residual lung damage detected by LUS could be responsible for a decrease in pulmonary parenchyma distensibility or static compliance [29]. It is possible that both, the low static compliance values, and most of the wounds observed by ultrasonography, indicate residual pulmonary injuries that return to normal as time passes.

In our study, the use of LUS identifies certain pulmonary alterations in asymptomatic post-COVID-19 patients undergoing surgery, and some of these pulmonary findings could be attributed to the SARS-CoV-2 since the patients did not present previous pulmonary pathology; despite this, the incidence of PPC is low. Most studies determine the PPC in patients with active SARS-CoV2 infection [30, 31]; in these cases, it is foreseeable that the complications will be greater than in those patients who have passed the infection or patients who have been asymptomatic. Other studies found an increase in PPC (51.2%) and mortality (23.8%) in patients who had SARS-CoV2 infection confirmed within 7 days before or 30 days after surgery [2]. However, the incidence obtained of PPC in our study was much lower, probably because our patients had an average number of days from diagnosis of SARS-CoV2 infection to surgery 108.56 ± 82.02 days, in the absence of symptoms and with negative PCR. These conditions would make surgery feasible. So, as we observed PPC in 5.8% of our patients; this incidence corresponds to values obtained in the pre-pandemic stage [32, 33], oscillating in some patients between 2 to 19% [34]. Furthermore, some authors, also, did not observe differences in their postoperative results when comparing SARS-CoV-2 positive patients with patients who had tested negative for SARS-CoV- 2 [16]. These authors argue that the baseline characteristics of the patients have an important impact in the development of PPC. Similarly, we observed that the baseline characteristics of the patients, evaluated according to the ASA classification, are associated with the appearance of PPC. Therefore, most of patients with PPC presented high ASA scores. Other factors, such as a severe evolution of the SARS-CoV-2 infection (in terms of pneumonia, PTE, the need for vasoactive drugs, hospital stay) and both, pleural thickening, and B lines ≥ 3, were also observed in our patients with PPC.

Our study has some limitations. Given the low number of complications, we only studied whether the influence of the pulmonary pathological findings and the severe course of the SARS-CoV-2 infection affect the appearance of any of the PPC, and not of each of the PPC separately. Also, regarding the LUS technique, as with many areas of ultrasound imaging and interpretation, the identification and quantification of the nature of B-lines and pleural line can be somewhat subjective and subject to interpretation [35]. To resolve this possible misinterpretation of LUS findings, these examinations could be performed by more than one expert anesthesiologist. Even by taking this limitation into account, it’s known that the LUS constitutes a useful predictive tool of clinical respiratory deterioration course and outcome, with the advantage that it can be easily performed bedside [36–40]. Moreover, the incidence of residual lung alterations that we observed in our study cannot be attributed completely to SARS-CoV-2 infection, since it is possible that these lesions are observed in patients without SARS-CoV-2 and without previous lung pathology.

## Conclusion

In asymptomatic post-COVID-19 patients undergoing surgery, the severity of SARS-CoV-2 infection in terms of hospitalization, need for mechanical ventilation, the clinical course of pneumonia, and the need for vasoactive drugs, is associated with an increase of pulmonary pathological findings determined by LUS. Nevertheless, the incidence of PPC could correspond to that described in the pre-pandemic stage.

## Data Availability

All data that support the findings of this study will be available from the corresponding author.

## Abbreviations

ARDS: Acute respiratory distress syndrome
ASA: American Society of Anesthesiologist
Cs: Static compliance
CT: Computed tomography
FiO2: Fraction of inspired oxygen
ICU: Intensive care medicine
IMV: Invasive mechanical ventilation
LUS: Lung ultrasound
NICE: National Institute for Clinical Excellence
NIMV: Non-invasive mechanical ventilation
PaO2: Partial pressure of oxygen
PEEP: Positive end expiratory pressure
PPC: Postoperative pulmonary complications
P plateau: Plateau pressure
PTE: Pulmonary thromboembolism
RNA: Ribonucleic acid
RT-PCR: Reverse-transcription polymerase chain reaction
Vt: Tidal Volume
